# Human atrial fibroblasts and their contribution to supraventricular arrhythmia

**DOI:** 10.14814/phy2.12711

**Published:** 2016-02-11

**Authors:** Morten B. Thomsen, Kirstine Calloe

**Affiliations:** ^1^ Department of Biomedical SciencesUniversity of CopenhagenCopenhagenDenmark; ^2^ Department of Veterinary Clinical and Animal ScienceUniversity of CopenhagenCopenhagenDenmark

**Keywords:** atrial fibrillation, electrophysiology, fibroblasts

The healthy heart is heavily populated with fibroblasts. The mouse heart contains 56% cardiomyocytes, 27% fibroblasts, 7% endothelial cells, and 10% vascular smooth muscle cells, based on a study where hearts were enzymatically digested and the cells immunolabeled and sorted according to fluorescence, FACS (Banerjee et al. 2007). Another study from rat heart reports only 30–35% cardiomyocytes and 65–70% nonmuscle cells (Nag 1980). Despite their high numbers, the fibroblasts constitute less than 25% of the mass of the heart, and the majority of the mass comprised cardiomyocytes (Vliegen et al. 1991). The relative size of the fibroblasts is small; cell sizes measured as electrical capacitance are approximately 15 pF for freshly isolated fibroblasts (Poulet et al. 2016) compared to 90 pF atrial cells and 190 pF of ventricular cells (Calloe et al. 2013). The cardiac fibroblasts secrete the majority of the extracellular matrix proteins, like collagens, laminin, proteoglycans, and fibronectin, and are pivotal for keeping the three‐dimensional structure of the heart.

Myofibroblasts are fibroblasts that are expressing actin and myosin. Fibroblasts are continuously adapting to their environment, and especially mechanical stimuli will modulate the expression of these contractile proteins. Not all agree on a sharp separation between the fibroblast and the myofibroblast and take the view that the fibroblast is a dynamic cell type that can express various amounts of actin and myosin, suggesting that the transition into myofibroblast is not a differentiation of the cell. Myofibroblasts are not found in the healthy heart, but they play a key role in the reparative fibrosis and scarring after myocardial infarction (Souders et al. 2009).

Fibroblasts secrete the extracellular matrix and can thereby affect cardiac electrophysiology by separating strands of cardiomyocytes with interstitial fibrosis. This causes a regionally reduced conduction velocity. Functional coupling between fibroblasts and cardiomyocytes has not been shown in human tissue; however, in animal models and cell cultures, it has been demonstrated that fibroblasts and cardiomyocytes can couple electrically to each other (Camelliti et al. 2004). This can affect the electrical properties of the cardiomyocytes, both passively and actively. The passive effects are due to the resistive and capacitive load on the cardiomyocyte. Fibroblasts are electrically nonexcitable (i.e., they do not make action potentials), but they have a high membrane resistance, and efficient coupling to a cardiomyocyte causes a dilution of the current density when membrane area increases without an increase in the number of ion channels. Moreover, fibroblasts may actively affect the action potential shape in cardiomyocytes (Camelliti et al. 2005; Kakkar and Lee 2010). Compared to cardiomyocytes, the fibroblasts have a less negative membrane potential (−50 to −10 mV; Kohl and Gourdie 2014), so when the fibroblasts are coupled to cardiomyocytes, depolarization of the cardiomyocytes will generate a flow of gap junctional current into the fibroblasts. This causes activation of voltage gated outward K^+^ currents in the fibroblasts, which may shorten the action potential in the cardiomyocyte. The less negative potential of the fibroblasts can in turn depolarize the coupled cardiomyocyte during diastole. The effect of this depolarization on cardiomyocyte excitability is complex. A slight depolarization can bring the potential closer to threshold for action potential firing, resulting in triggered activity, but will more likely result in sodium channel inactivation and thereby slow depolarization during the action potential. The increased capacitive load after coupling will also contribute to diluting the depolarization force of the cardiomyocyte. Thus, the fibroblasts may be an important component of the cardiac syncytium in disease, where fibroblast–cardiomyocyte coupling will reduce conduction velocity and potentially render the cardiomyocytes unexcitable. The impact of fibroblasts on cardiac electrophysiology depends on (1) how well the cells are coupled; and (2) the electrical properties of the fibroblast. Yet, all these parameters are not fully investigated in vivo, and the functional importance of fibroblast–cardiomyocyte coupling still needs to be established in both healthy and diseased human hearts.

Dr. Poulet and colleagues have elegantly probed the electrophysiological properties of human atrial fibroblasts from patients in normal sinus rhythm and in chronic atrial fibrillation (Poulet et al. 2016). Atrial fibrillation is the most common cardiac arrhythmia and it significantly affects morbidity and mortality. Just over half of the isolated fibroblasts were characterized as myofibroblasts and this fraction was comparable in sinus rhythm and atrial fibrillation biopsies. However, significantly fewer cells were obtained from atrial fibrillation samples, and during culture of the isolated cells, the fibroblasts from patients with atrial fibrillation proliferated less and had lower migratory activity. Next, the authors not only measured ion currents in fresh fibroblasts, but also cultured the fibroblasts to probe the degree of fibroblast remodeling ex vivo. The membrane capacitance was not different in freshly isolated sinus rhythm and atrial fibrillation fibroblasts. A large time‐dependent remodeling in both cell sizes as well as electrophysiological properties was noted after culture. For freshly isolated atrial fibroblasts, there were tendencies for larger inward and outward currents in cells from patients with atrial fibrillation; however, current amplitudes were substantially variable in the freshly isolated cells and the differences did not reach statistical significance. The authors suggest that nonselective cation TRP channels could be responsible for the currents observed in the freshly isolated fibroblasts, and it has previously been shown that TRP channels have a role in the activation of fibroblasts and they are upregulated in fibroblasts from fibrillating atria (Du et al. 2010; Harada et al. 2012).

Why were the fibroblast yield smaller and their proliferative capacity poorer when the samples came from fibrillating atria? Poulet et al. report significant differences in Na^+^ and K^+^ currents in sinus rhythm and atrial fibrillation fibroblasts after weeks in culture (Poulet et al. 2016), but how does this relate to fibroblast remodeling in situ? These questions highlight the need for more studies of the electrophysiological properties of freshly isolated atrial fibroblasts.

How can insights into fibroblast electrophysiology help us understand the progression of atrial fibrillation and suggest novel treatment modalities? Focal ectopy and re‐entry are the arrhythmogenic mechanisms underlying AF initiation and maintenance. The presence of atrial fibrillation, even for a short time period, significantly affects the atrial electrical properties, augmenting the likelihood of both focal activity and re‐entry. Thus, atrial fibrillation promotes more atrial fibrillation, until the brief bouts of arrhythmia becomes chronic atrial fibrillation (Wijffels et al. 1995). Both the presence of fibroblasts and the presence of fibrosis in the atria have impact on conduction velocity in the atria. Reduced conduction velocity sets the stage for re‐entrant circuits making atrial fibrillation all the more likely. However, it is clear that fibroblasts and fibrosis in the atria promotes atrial fibrillation, it is much more unclear whether the opposite takes place: Does atrial fibrillation promote the proliferation of fibroblasts, coupling to myocytes and deposition of fibrosis?

The physiological importance of the electrical coupling between cardiomyocytes and fibroblasts still needs to be established in patients. It is important to gain a better quantitative and qualitative understanding of the electrophysiology of freshly isolated fibroblasts as well as the strength of coupling between fibroblasts and cardiomyocytes in vivo. Weak coupling to a single fibroblast may have a negligible effect, whereas strong coupling to multiple fibroblasts could have a large effect on the electrophysiology of the cardiomyocyte. Both electrophysiology of fibroblasts as well as coupling strength could be affected by disease and the fibroblast proliferation, activity, as well as the coupling between fibroblasts and cardiomyocytes may offer potential new targets for pharmaceutical intervention.

## Conflict of Interest

None.
